# The human P2X7 receptor alters microglial morphology and cytokine secretion following immunomodulation

**DOI:** 10.3389/fphar.2023.1148190

**Published:** 2023-04-10

**Authors:** Iven-Alex von Mücke-Heim, Jana Martin, Manfred Uhr, Clemens Ries, Jan M. Deussing

**Affiliations:** ^1^ Molecular Neurogenetics, Max Planck Institute of Psychiatry, Munich, Germany; ^2^ Core Unit Analytics and Mass Spectrometry, Max Planck Institute of Psychiatry, Munich, Germany

**Keywords:** P2X7 receptor (P2X7R), mouse model, cell culture, immunomodulation, knockout (KO) mice, microglia, cytokine secretion

## Abstract

**Introduction:** In recent years, purinergic signaling via the P2X7 receptor (P2X7R) on microglia has repeatedly been implicated in depression genesis. However, it remains unclear which role the human P2X7R (hP2X7R) plays in regulating both microglia morphology and cytokine secretion upon different environmental and immune stimuli, respectively.

**Methods:** For this purpose, we used primary microglial cultures derived from a humanized microglia-specific conditional P2X7R knockout mouse line to emulate different gene-environment interactions between microglial hP2X7R and molecular proxies of psychosocial and pathogen-derived immune stimuli. Microglial cultures were subjected to treatments with the agonists 2′(3′)-O-(4-benzoylbenzoyl)-ATP (BzATP) and lipopolysaccharides (LPS) combined with specific P2X7R antagonists (JNJ-47965567, A-804598).

**Results:** Morphotyping revealed overall high baseline activation due to the in vitro conditions. Both BzATP and LPS + BzATP treatment increased round/ameboid microglia and decreased polarized and ramified morphotypes. This effect was stronger in hP2X7R-proficient (CTRL) compared to knockout (KO) microglia. Aptly, we found antagonism with JNJ-4796556 and A-804598 to reduce round/ameboid microglia and increase complex morphologies only in CTRL but not KO microglia. Single cell shape descriptor analysis confirmed the morphotyping results. Compared to KO microglia, hP2X7R-targeted stimulation in CTRLs led to a more pronounced increase in microglial roundness and circularity along with an overall higher decrease in aspect ratio and shape complexity. JNJ-4796556 and A-804598, on the other hand, led to opposite dynamics. In KO microglia, similar trends were observed, yet the magnitude of responses was much smaller. Parallel assessment of 10 cytokines demonstrated the proinflammatory properties of hP2X7R. Following LPS + BzATP stimulation, IL-1β, IL-6, and TNFα levels were found to be higher and IL-4 levels lower in CTRL than in KO cultures. Vice versa, hP2X7R antagonists reduced proinflammatory cytokine levels and increased IL-4 secretion.

**Discussion:** Taken together, our results help disentangle the complex function of microglial hP2X7R downstream of various immune stimuli. In addition, this is the first study in a humanized, microglia-specific *in vitro* model identifying a so far unknown potential link between microglial hP2X7R function and IL-27 levels.

## 1 Introduction

Major depression is a common and multifactorial mental disorder, which arises from complex gene-environment interactions (GBD-2017-Collaborators, 2018; Lopizzo et al., 2015). Alarmingly, current trend analyses report a significant and worldwide rise in depression incidence (Moreno-Agostino et al., 2021). Aside from the well characterized impact of genetic vulnerability and acute as well as chronic psychosocial stress (Lopizzo et al., 2015; Gonda et al., 2018), alterations in cellular and humoral immunity feeding into mild systemic inflammation have been identified as important factors in depression genesis (Miller and Raison, 2016; Wohleb et al., 2016; Medina-Rodriguez et al., 2018), at least in a subset of about 30% of patients (Raison and Miller, 2011; Nobis et al., 2020). The latter experience elevated blood levels of proinflammatory cytokines like tumor necrosis factor alpha (TNFα), C-reactive protein (CRP), or interleukin (IL)-6 (Felger and Lotrich, 2013; Miller and Raison, 2016; Mac Giollabhui et al., 2020), changes in immune cell composition and function (Miller, 2010; Miller and Raison, 2016; Drevets et al., 2022), which are often accompanied by altered metabolic parameters (de Kluiver et al., 2019; Lamers et al., 2020). Especially the monocyte-macrophage system including microglia, natural killer cells (NKs), and regulatory T cells (T_reg_) along with different T helper cells (Th) display molecular and functional aberrations in depressed patients (Miller, 2010; Suzuki et al., 2017; Beurel et al., 2020; Cattaneo et al., 2020; Bauer and Teixeira, 2021). A recent study by Costi and colleagues, for instance, demonstrated a relation between elevated blood-derived immune cell reactivity in the form of cytokine release following *ex vivo* lipopolysaccharides (LPS) stimulation and anhedonia as well as reward anticipation in depression (Costi et al., 2021). This aligns with the existing literature on the relation between cytokine burden and symptom load (Rengasamy et al., 2021; Roohi et al., 2021; Sha et al., 2022), as well as with the beneficial effects of different anti-cytokine treatments for depressive symptoms in systemic inflammatory disorders (Köhler et al., 2014; Kappelmann et al., 2018; Wittenberg et al., 2020). This highlights the importance of immune mechanisms in psychiatric disorders and the need to improve our understanding of its complex interplay with depression genesis, clinical course, and symptom burden (Himmerich et al., 2019).

Among the different immune pathways potentially involved in depression genesis, purinergic signalling *via* the adenosine triphosphate (ATP) selective, membrane bound ionotropic P2X7 receptor (P2X7R) is considered a promising candidate (Di Virgilio et al., 2017; Deussing and Arzt, 2018; Drevets et al., 2022). Multiple studies have shown its involvement in stress-induced inflammation, depression, and depressive-like behaviours (von Mücke-Heim and Deussing, 2022; von Muecke-Heim et al., 2021). In the context of mood disorder genesis (Andrejew et al., 2020; Illes et al., 2020), both clinical and preclinical findings from mouse models emphasize the role of chronic psychosocial stress in causing ATP release in the central nervous system (CNS), leading to sterile inflammation *via* P2X7R-depedent purinergic pathways, which contribute to the development of depression (Franklin et al., 2018; Iwata et al., 2016; Ribeiro et al., 2019a; Velasquez and Rappaport, 2016; von Mücke-Heim and Deussing, 2022; Wohleb et al., 2016). On a functional level, binding of ATP or its synthetic agonist 2′(3′)-O-(4-benzoylbenzoyl)-ATP (BzATP) trigger rapid potassium efflux and sodium plus calcium influx, initiating proinflammatory intracellular pathways including, but not limited to, the NLR family pyrin domain containing 3 (NLRP3) inflammasome (Adinolfi et al., 2018). Downstream of these pathways, cells release proinflammatory cytokines like IL-1β, IL-6, and TNFα (Di Virgilio et al., 2017; Junger, 2011; von Muecke-Heim et al., 2021). Aptly, P2X7R is considered a major player in inflammation (Di Virgilio et al., 2017; Adinolfi et al., 2018). It is expressed on immune cells and is functionally particularly important for microglia and T cells (Junger, 2011; Jacobson and Müller, 2016; He et al., 2017; Burnstock, 2018; Rivas-Yáñez et al., 2020). In microglia and monocytes, P2X7R activation has differential effects on M1 and M2 states (de Torre-Minguela et al., 2016; Jiang et al., 2022). Still, it remains largely unknown which exact role the human P2X7R (hP2X7R) on microglia plays in regulating morphology and cytokine expression after P2X7R-targeted stimulation or inhibition.

The present study therefore aims to shed light on these mechanisms by use of primary microglia cultures of a humanized microglia-specific conditional knockout mouse line (hP2RX7-Cx3cr1) (Metzger et al., 2017). A cluster of pro- and anti-inflammatory cytokines along with microglial morphology were analysed at baseline as well as following both P2X7R agonistic and antagonistic stimuli. To the best of our knowledge, this is the first study to examine hP2X7R-expressing murine microglia beyond the well characterized IL-1β and TNFα release on a morphological as well as secretory level.

## 2 Materials and methods

### 2.1 Animals

Animals were housed in individually ventilated cages (Tecniplast, IVC Green Line-GM500) under standard laboratory conditions (21°C ± 2°C, 50% ± 5% humidity, 12:12 h light:dark cycle, lights on at 7:00 a.m.). Food and water were provided *ad libitum*. All experiments and protocols were performed in accordance with the European Communities’ Council Directive 2010/63/EU and were approved by the committee for the Care and Use of Laboratory animals of the Government of Upper Bavaria, Germany.

Humanized mice expressing the human P2X7R (hP2X7R) instead of the murine variant had been established earlier (*P2rx7* ^*tm1.1(P2RX7)Jde*^). The humanized *P2rx7* allele is additionally equipped with *loxP* sites which allow for conditional disruption of hP2X7R expression (Metzger et al., 2017). Conditional knockout mice lacking hP2X7R in microglia were generated by breeding homozygous floxed hP2X7R mice (*hP2rx7* ^*P2RX7/P2RX7*^) to the microglia-specific tamoxifen-inducible driver line *Cx3cr1-CreERT2* (*Cx3cr1* ^*tm2.1(cre/ERT2)Litt*^; Parkhurst et al., 2013). Mice for primary cultures were obtained from breeding *hP2rx7* ^*P2RX7/P2RX7*^ *; Cx3cr1* ^*+/+*^ females to *hP2rx7* ^*P2RX7/P2RX7*^ *; Cx3cr1* ^*+/CreERT2*^ males. Control (CTRL; *hP2rx7* ^*P2RX7/P2RX7*^ *; Cx3cr1* ^*+/+*^) and conditional knockout mice (KO; *hP2rx7* ^*P2RX7/P2RX7*^ *; Cx3cr1* ^*+/CreERT2*^) were born in a 1:1 ratio. Genotyping was performed by polymerase chain reaction (PCR) analysis of tail DNA using specific primers. *P2rx7:* hP2X7R-mIntron1-for 5′-AGA CTG TCA CCA GCA GCA GCT C-3′, hP2X7R-mIntron2-rev-a 5′-GCC AAG CAT TCT ACC AGT TGA-GC-3′, hP2X7R-hExon2 rev 5′-CAC GAA GAA AGA GTT CCC CTG C-3′, hP2X7R-KO-for 5′-GCA GTC TCT CTT TGC CTC GT-3′, hP2X7R-KO-rev 5′-CGT CGA CTG TCT TCT GGT CA-3′ (PCR products: wildtype = 417 bp; floxed = 298 bp; knockout = 222 bp). *Cx3cr1*: Cx3cr1-Cre-14278 5′-CTC CCC CTG AAC CTG AAA C-3′, Cx3Cr1-Cre-14277: 5′-CCC AGA CAC TCG TTG TCC TT-3′, Cx3Cr1-Cre-14276 5′-GTC TTC ACG TTC GGT CTG GT-3′ (PCR products: wildtype = 500 bp, Cre = 410 bp).

### 2.2 Primary microglia culture

Preparation of primary murine microglia cultures was performed as previously described (Lian et al., 2016). Standard T75 cell culture flasks (Sarstedt) were coated with 0.05 mg/mL poly-D-lysine (PDL, 70.000 mol wt, Sigma Aldrich) followed by three washing steps. Pups were decapitated at P1–P3 and the heads were immediately transferred to ice-cold dissection medium Dulbecco’s Modified Eagle’s Medium F-12 Nutrient Mixture (DMEM/F-12) with GlutaMax™ supplement (Gibco™, Sigma Aldrich) and 1% Penicillin/Streptomycin (P/S) (Fisher Scientific). Brains were extracted on a horizontal-laminar flow bench under sterile conditions. After the meninges, brainstem, cerebellum, and white matter were removed, the remaining cortical tissue was reduced to small pieces by use of microsurgical scissors, followed by glass Pasteur pipette dissociation. The suspension was centrifuged for 5 min at room temperature (RT) at 1,200 rpm. Supernatant was discarded and neuroglial cells were seeded in PDL coated flasks in dissection medium plus 10% heat-inactivated fetal bovine serum (Merck), 1% non-essential amino acids (Gibco™, Fisher Scientific), and 1% sodium pyruvate (Gibco™, Fisher Scientific). Individual neuroglia cultures were maintained for 14 days with two medium changes per week.

At day 15, microglia were extracted from neuroglia culture flasks by combined enzymatic and mechanic separation. In accordance with Saura et al. (2003) and Caldeira et al. (2014), flasks were first incubated for 30–60 min at 37°C with 0.25% Trypsin-EDTA (Gibco™, Fisher Scientific) diluted 1:2 with dissection medium (Saura et al., 2003; Caldeira et al., 2014). This mild trypsinization caused the detachment of a carpet-like layer of cells containing mainly astrocytes, leaving microglia attached to the flask bottom. In a second step and adapted from Woolf et al. (2021), pure 0.25% Trypsin-EDTA was added to the flask for 5 min (Woolf et al., 2021). In the meantime, flasks were shaken and tapped gently, resulting in an almost complete detachment of microglia. Trypsin-EDTA was then blocked by addition of serum-containing growth medium. Cells were centrifuged for 5 min at RT at 1,200 rpm and supernatant was discarded. Quantification of cells was performed by use of the Trypan Blue (Gibco™, Fisher Scientific) exclusion test using a Neubauer counting chamber. At least two 16-square quadrates were counted per flask (Strober, 2015). In the end, 5 × 10^4^ cells were seeded per well in 0.5 mL growth medium in 24-well plates equipped with PDL-coated 5 mm cover slips.

Cre-mediated *hP2RX7* inactivation was induced by 4-hydroxytamoxifen (OH-TAM, Sigma Aldrich). OH-TAM was dissolved in methanol and added (final concentration 1 µM/well). OH-TAM treatment lasted 7 days and included two growth medium changes.

### 2.3 Treatments with LPS, BzATP, and selective P2X7R antagonists

To emulate different gene-environment interactions between microglial hP2X7R and molecular proxies pf psychosocial and pathogen-derived immune challenges, cell cultures wells were subjected to the following treatments at day 22 after the initial brain extraction: either 1) 30 min of 1 µM JNJ-47965567 (Sigma Aldrich), 2) 30 min of 10 µM A-804598 (Sigma Aldrich), 3) 60 min of 200 μM BzATP (Sigma Aldrich), or 4) priming for 24 h with 100 ng/mL LPS (Sigma Aldrich) plus 60 min of 200 μM BzATP. LPS and BzATP were dissolved in water and JNJ-47965567 along with A-804598 were solved in pure dimethyl sulfoxide (DMSO, Cell Signaling Technology). The added volume of all treatment substances per well was chosen as small as practically feasible (i.e., 0.49 µL of the JNJ-47965567 stock solution in 500 µL medium ≙ 0.1 %v/v; 3.2 µL of the A-804598 stock solution in 500 µL medium ≙ 0.6 %v/v) to avoid DMSO-related cytotoxicity. For each animal, two separate cell culture wells remained untreated as controls and were both used for analysis. Concentrations of stimulations and selective P2X7R antagonists were chosen based on previous studies to model a solitary stress-related (i.e., stimulation with BzATP) or double hit in the form of a bacterial plus stress-related stimulus (i.e., LPS + BzATP) *in vitro* (Bradley et al., 2011; de Torre-Minguela et al., 2016; He et al., 2017; He et al., 2021). In the end, cell culture supernatant was aliquoted in PE tubes and stored at −20°C for cytokine measurements. The two control wells per animal were pooled to minimize variability.

### 2.4 Cytokine measurements

To study the effects of different immunomodulatory stimuli on microglial hP2X7R-dependant cytokine release, the post-treatment cell culture supernatants were analyzed. The following cluster of pro- and anti-inflammatory cytokines was selected to, among other things, allow inferences for leucocytes, NK and T cells, and microglia with their proinflammatory M1 and anti-inflammatory M2 pole (Frade and Barde, 1998; Zhang and An, 2007; Zhu et al., 2010; Orihuela et al., 2016; Dubbelaar et al., 2018; Martinez-Sanchez et al., 2018; Himmerich et al., 2019; Jurga et al., 2020): TNFα, INF-γ, IL-1β, IL-2, IL-4, IL-6, IL-10, IL-12, IL-17/IL-17A, IL-27. To measure these factors *in vitro*, a custom-made high-sensitivity analyte kit for murine cytokines was purchased from R&D Systems (Luminex^®^ Discovery Assay).

Before measurements were performed, the Luminex^®^ 200 instrument (Invitrogen) was cleansed and calibrated according to its operating instructions. Sample preparation and measurements were performed strictly according to the kit manufacturer’s specifications. In brief, the provided standards were reconstituted to establish two separate serial dilutions (i.e., 6 times 10 × dilution). Growth medium and calibrator diluent were added to improve the signal-to-noise ration by correcting for fluorescent background noise caused by the different carrier substances. Samples were mixed 1:1 (=1:2 dilution) with the calibrator diluent (75 µL each). Next, either 50 µL of samples and standard was transferred to the 96-well plate and 50 µL of fluorescent bead cocktail was added. The plate was covered with aluminum foil and incubated at RT on a horizontal shaker at 800 rpm for 2 h. Wells were cleansed 3x with wash buffer using a magnetic plate clipped to the 96-well plate bottom. Next, 50 μL of diluted biotin-antibody cocktail was added to each well and incubated in the dark at RT on a shaker at 800 rpm for 1 h. Wells were washed again 3x, followed by the addition of 50 μL of diluted Streptavidin-phycoerythrin and a 30 min incubation period at RT on a shaker at 800 rpm. Washing was performed another 3x and 100 μL of wash buffer was added to each well, followed by 2 min at RT on a shaker at 800 rpm. Finally, the 96-well plate was then transferred to the Luminex^®^ 200 system and measurements were commenced. Doublet discriminator gates were set at 8,000 and 16,500, while reporter gain settings were left at default. For quality control, triplicate measurements were performed in two independent samples with different activation levels (i.e., LPS + BzATP in a Cre-negative sample; control sample of a Cre-positive animal). Results confirmed reliability of measures (Supplementary Figure S1). Standard curves were calculated by use of five parameters logistic regression and the provided six standards. Based on these, cytokine concentrations (pg/mL) were extracted and values were then doubled due to the 1:2 dilution. Next, the background (i.e., mean cytokine concentration of the two untreated growth medium measurements) was subtracted. Since in some groups control wells yielded a concentration of 0 pg/mL, each treatment well’s value was put into relation to the mean of the group’s control wells (*x = individual value treatment/mean of group’s control wells*). The controls contained the cell culture supernatant of the two control wells per animal in a 1:1 mixture.

### 2.5 Immunocytochemistry und fluorescence microscopy

Cover slips were fixed with 4% paraformaldehyde for 30 min, washed 3x with phosphate buffered saline (PBS) and then blocked with 5% normal goat serum (NGS, Thermo Fisher Scientific) in 0.2% Triton X-100 PBS for 30 min. Primary antibody incubation with rabbit anti-Iba1 (Synaptic Systems), rat anti-CD86 (Invitrogen), or mouse anti-cleaved-caspase 3 (Affinity) was performed over night with a 1:500 dilution under mild agitation at 4°C in the dark. The next morning, cover slips were washed 3x with PBS. Secondary antibody incubation was done using 1:500 goat anti-rabbit Alexa Fluor 488 (Invitrogen) or anti-rat Alexa Fluor 488 (Invitrogen) in 5% NGS in 0.2% Triton X-100 PBS at RT and under mild agitation in the dark for 2 h. Lastly, cover slips were washed thrice with PBS and mounted on standard glass slides with 20 µL of DAPI Fluoromount-G™ (Invitrogen).

Fluorescence images were acquired on an Olympus VS120 Slide Scanner. Per cover slip, two regions of interest (ROI, size: ∼1 mm^2^) were chosen at random in the DAPI channel to avoid bias based on microglia morphology to calculate cell numbers. The same ROIs were used for all image analyses and the respective statistics. Images were taken with a ×20 magnification, an exposure time of 515 ms (Iba-1/Alexa488 channel) and a Z-range of 18 µm (spacing: 6 µm, plane count: 3). Experimenters were blinded to genotype and treatments during image acquisition and analyses.

### 2.6 Image analysis: Cell numbers and microglia morphology

**FIGURE 1:**
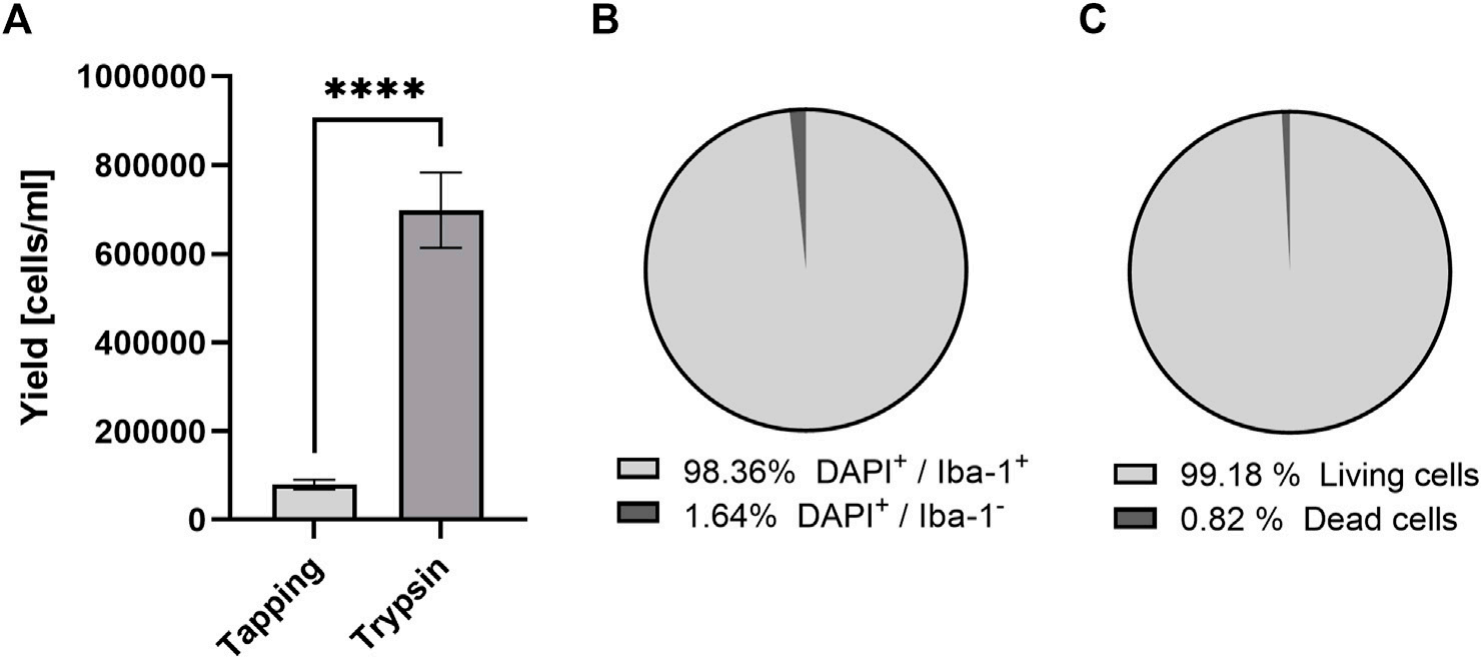
Purity and vitality of microglia based on two different isolation methods irrespective of genotype. Comparison of microglia yield from tapping and two-step mild trypsinization: **(A)** The total number of cells, **(B)** the proportion of microglia and **(C)** vital cells after 14 days of culturing after microglia enrichment. Data are expressed as mean ± S.E.M, T-test with Welch correction; tapping: *n* = 33, mild trypsinization: *n* = 25; *n* = 10 ROIs.

Images were analyzed using the Fiji software package (GNU General Public License, Release 2.9.0). Microglia number were calculated using a custom-designed macroinstruction (macro). Due to the purity of our cultures (Figure 1), a double discriminatory approach was unnecessary. Automated cell counting was therefore performed in the DAPI channel. The generated Fiji macro was validated, yielding no difference between the manual quantification of two blinded and independent experimenters and the automated macro results (Supplementary Figure S2).

Since microglia are highly reactive and dynamic cells, they respond to an array of stimuli by changes in their morphology. The latter can thus serve as a proxy of the underlying genetic, molecular, and functional changes as well as the environmental circumstances (Nimmerjahn et al., 2005; Dubbelaar et al., 2018; Fernández-Arjona et al., 2019; Jurga et al., 2020). Microglia morphology analysis was based both on morphotype categories and objective shape descriptors. Based on previous observations from our lab and in the style of a related study by He et al. (2021), microglia were classed by the respective experimenter according to three distinct morphotypes: 1) round or amoeboid (no processes), 2) polarized or rod-like (length-to-width ratio ≥ 3), and ramified (≥3 extensions/ramification). To obtain objective measures of cell shape, maximum intensity projections (MIP) containing both DAPI (blue) and Iba-1/Alexa488 (green) were generated and microglia were manually delineated. The following shape descriptors were extracted for each individual cell:

$$Circularity=4\pi *\left(\frac{Area}{Perimeter^{2}}\right)\;Aspect\;ratio=\frac{Major\;axis}{Minar\;axis}$$

$$Roundness=4*\frac{Cell\;area}{\left(\pi *major\;axis^{2}\right)}\;Solidity=\frac{Cell\;area}{Convex\;area}$$

Based on morphological assumptions (i.e., resting microglia have a small soma and many, thin, and long ramifications; activated microglia loose or reduce ramifications and their soma enlarges), we devised the following complexity index:

$$Complexity\;index=\frac{Perimeter}{Cell\;area}$$

For circularity and solidity, the interval of potential values is x ∈ [0; 1]. The interval for the aspect ratio and complexity index is x ∈ [0; ∞]. As an *in vitro* alternative to the often used skeletonization approach in immunohistochemistry (Morrison et al., 2017; Young and Morrison, 2018), we developed the aforementioned complexity index. It rests on the assumption, that microglia are closer to the *in vivo* resting state and that their shape complexity is higher, if the cell perimeter is long and the surface area is comparatively small. This means, the higher the complexity index, the more complex the cell. Within this hypothesis frame, rod-like or polarized microglia are also labeled as more complex than round or ameboid cells. In addition to the shape descriptors and indices, the cell area and perimeter were extracted.

For qualitative intensity analyses, mean cellular gray values were extracted from Iba1+/DAPI+ cells (i.e., microglia) in the Iba1/GFP-channel of both ROIs.

### 2.7 Statistical analysis and Z scoring

Data are expressed as mean ± standard error of the mean (SEM) or percentage. Statistical analysis was performed using GraphPad Prism (Version 9.3.1). Statistical significance was defined as *p* < 0.05 and indexed as follows: *p < 0.05, **p < 0.01, ***p < 0.001, and ****p < 0.0001. Depending on the sample size and data structure, analyses were performed by a two-tailed T-test with Welch correction, a Chi^2^ test, a one-Way ANOVA with Turkey’s multiple comparisons test, or the Kruskall-Wallis test (KW) followed by Dunn’s test for multiple comaprisons. To account for possible batch effects and experimental variability, cell numbers and shape descriptor data were put in relation to their two individual control wells [*x = individual value treatment/mean of control wells* (n-fold) OR *x = treatment value − mean of control wells* (delta)]. Morphological data were trimmed and outliers were calculated from raw values for each analysis type and excluded from group statistics if divergence was smaller than the first quartile (Q_1_) − 1.7 * interquartile range (IQR) or larger than Q3 + 1.7 * IQR. During data collection and analysis, experimenters remained blinded.

To allow a comparison of cytokine clusters between groups, Z tests and scores were used as previously described (Guilloux et al., 2011; von Mücke-Heim et al., 2022):

$$Z\;test=\frac{\left(X-\mu \right)}{\sigma }\;Z\;score=\frac{\left(Ztest1+Ztest2+Ztest3\right)}{Number\;of\;tests}$$

In brief, Z test calculations were performed for each cytokine and condition using hP2X7R KO sample values (X) and the CTRL groups mean (µ) and standard deviation (σ) of the same cytokine. Pro- and anti-inflammatory cytokine Z test results were then pooled in pro- and anti-inflammatory Z scores per animal and treatment condition.

To validate the cytokine Z score results, shape descriptor-based Z scores with similar directionality derived from roundness for pro-inflammatory and aspect ratio for anti-inflammatory clusters were added. As described above, hP2X7R KO sample values (X) and the CTRL groups mean (µ) and standard deviation (σ) of the respective shape descriptor were used to obtain shape descriptor Z scores. Lastly, the cytokine and shape descriptor Z scores were then summarized into a pro-inflammatory (ProIF) or anti-inflammatory (AntiIF) combined Z score per animal as follows:

$$ProIF\;Z\;score=\frac{Z\;score\;\left(IL-1,IL-6,TNF\right)+Z\;score\;\left(roundness\right)}{2}$$

$$AntiIF\;Z\;score=\frac{Z\;score\;\left(IL-4,IL-10\right)+Z\;score\;\left(aspect\;ratio\right)}{2}$$

## 3 Results

### 3.1 Mild trypsinization yields high-quality microglia cultures

**FIGURE 2:**
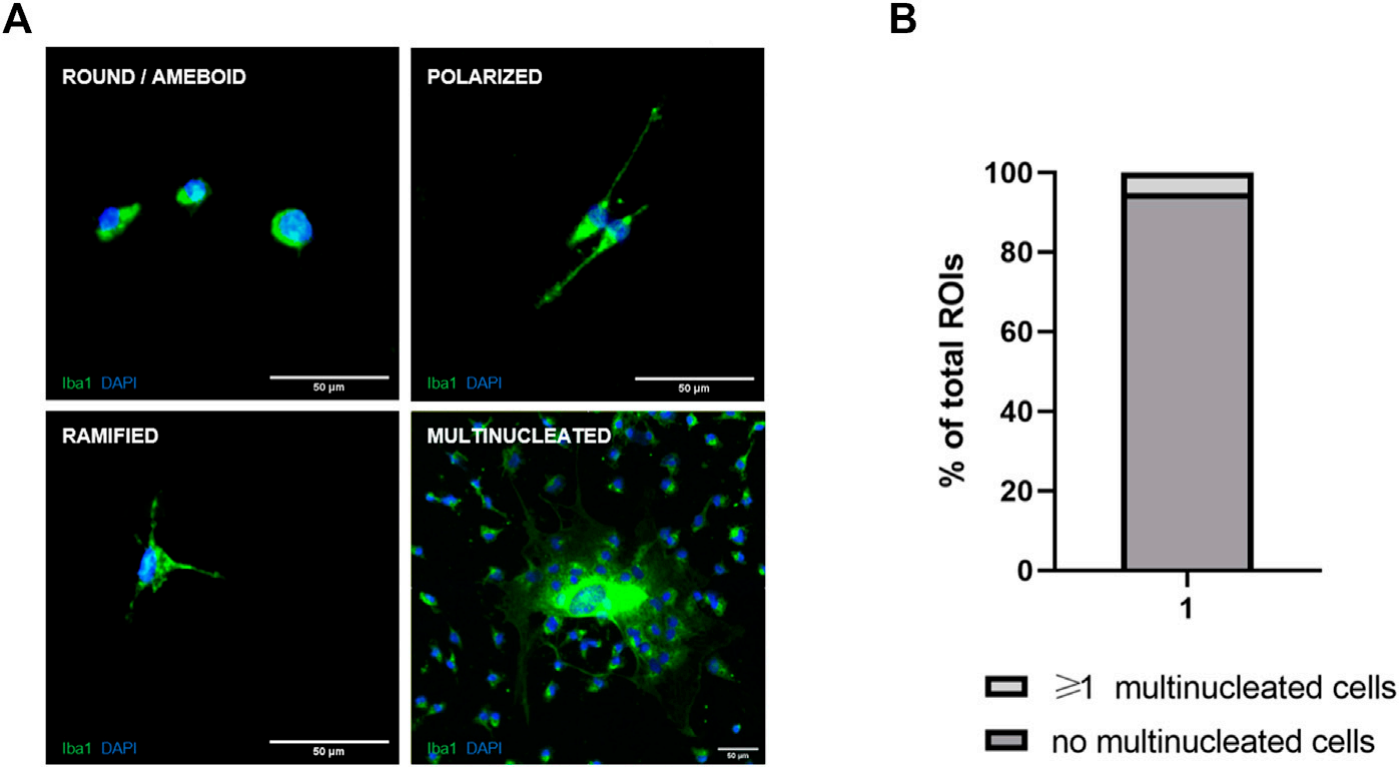
Morphotypes of microglia in primary cell cultures. **(A)** Representative images of the four main microglia morphotypes identified in primary cultures. **(B)** Percentage of ROIs with multinucleated Iba1-positive microglia in all samples irrespective of genotype.

We used the two-step mild trypsinization approach to isolate primary microglia (Saura et al., 2003; Woolf et al., 2021). In comparison to the shaking and tapping method (Lian et al., 2016), mild trypsinization yielded almost 9-fold more cells (Figure 1A). With the two-step trypsinization protocol we obtained cultures of high purity (≥98% of cells were microglia) and vitality (Figures 1B, C). Cultured microglia appeared healthy and, as known from primary murine cultures with serum-containing medium, less ramified than in the *in vivo* situation. As previously described (He et al., 2021), we were also able to identify three distinct and different microglia morphotypes. In addition, we detected large and multinucleated cells (Figure 2A). However, since multinucleated cells were present only in about 5% of all ROIs, quantitative statistical analysis was not done (Figure 2B).

### 3.2 hP2X7R evokes changes of microglia numbers and morphology

**FIGURE 3:**
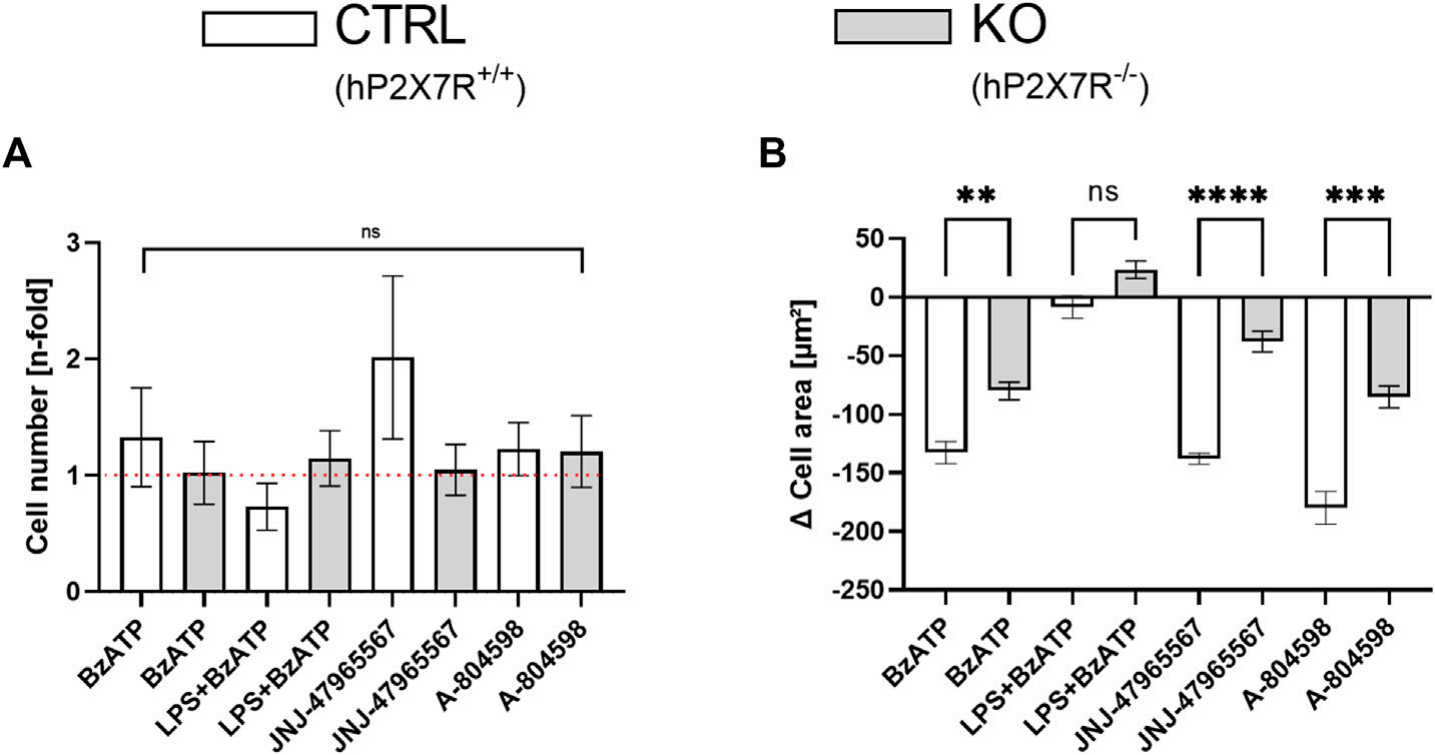
Cell count and area dynamics following hP2X7R-targeting treatments (i.e., BzATP, LPS + BzATP, JNJ-47965567, or A-804598). **(A)** Comparison of microglia cell number changes in relation to untreated genotype-matched control wells (visual red reference line: 1 = no difference) after treatment between CTRL and KO microglia. Cell counts were corrected per group by the respective untreated within-genotype controls. The hP2X7R-proficient group (CTRL) is displayed in white, the hP2X7R-knockout group (KO) is shown is grey. Data are expressed as mean + S.E.M, KW with Dunn’s test, *n* = 7–10 mice per group (A-804598 in hP2X7R wildtype animals *n* = 4). **(B)** Between-genotype comparison of microglia cell area changes following pro- and anti-inflammatory treatments. Cell areas were corrected per group by the respective untreated within-genotype controls. The hP2X7R-proficient group (CTRL) is displayed in white, the hP2X7R-knockout group (KO) is shown is grey. Data are expressed as mean + S.E.M, KW with Dunn’s test, *p < 0.05, **p < 0.01, ***p < 0.001, and ****p < 0.0001, *n* = 8–11 mice with 813–1989 cells per group (except: A-804598 in CTRL group, *n* = 5 mice and 654 cells).

At baseline, cell counts did not differ between microglial cultures isolated from hP2X7R proficient (CTRL) and P2X7R deficient (KO) animals, respectively (79 ± 61/µm^2^ in CTRLs and 82 ± 57/µm^2^; *p* > 0.05 in T-test with Welch correction). Following stimulation with BzATP, cell numbers increased by 33% in CTRL but not in KO cultures (Figure 3A). Whereas, the combined LPS + BzATP stimulation resulted in a 27% decline in CTRL and a minimal incline in KO cultures (Figure 3A). To uncover the reason for these observations, immunohistochemistry was performed: mean cellular intensity revealed higher values for cleaved caspase-3 and CD86 in LPS + BzATP treated CTRL compared to KO cultures (Supplementary Figure S3). This suggests more M1-polarization and apoptotic activity in double stimulated CTRL compared to KO cultures and a direct contribution of hP2X7R. The P2X7R antagonist-treated groups, on the other hand, showed higher cell counts in both genotypes. The difference was most prominent in JNJ-47965567-treated CTRL cells with a two-fold increment compared to controls. This increase, however, was mostly driven by one culture (5.3-fold gain), which also explains the high standard error in this group. Overall, changes in microglia numbers did not reach statistical significance (Figure 3A). The cell area, on the other hand, was significantly different in CTRL and KO animals in all treatment groups expect in the LPS + BzATP treated wells (Figures 3A, B). Mean indexed cell size decreased in the BzATP group as well as in the P2X7R antagonist conditions in both genotypes, but the extent of the decrease was more pronounced in CTRL cultures. This is interesting, since the absolute cell area mean was about 20% lower in CTRL microglia already at baseline (448 ± 210 μm^2^ in CTRLs and 553 ± 243 μm^2^).

**FIGURE 4:**
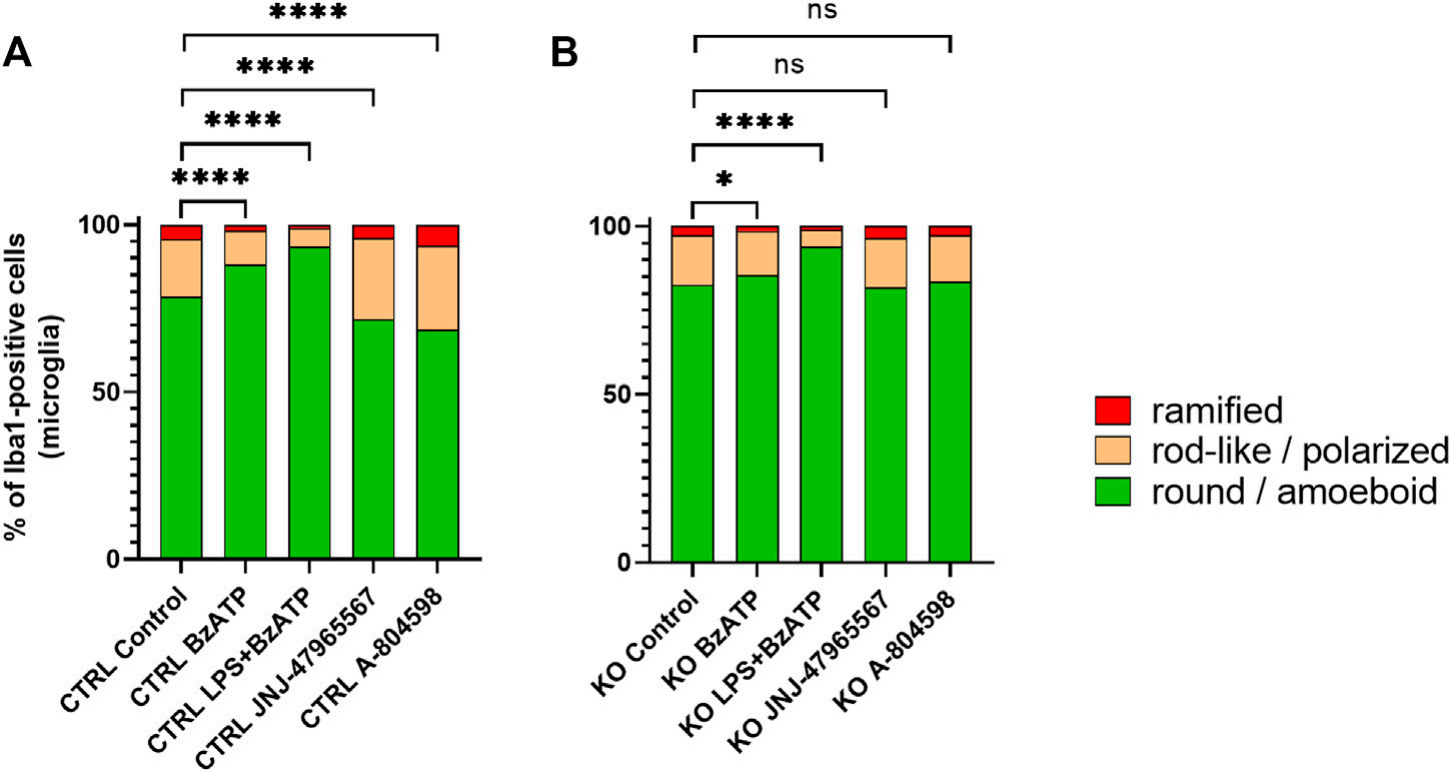
Manual morphotyping of microglial phenotype. Within-group comparison of **(A)** CTRL and **(B)** KO microglia morphology proportion changes between the baseline and after hP2X7R-targeting treatments (i.e., BzATP, LPS + BzATP, JNJ-47965567, A-804598). Data are expressed as % of total Iba1-positive cells per genotype and treatment condition including the untreated genotype controls. Chi^2^ Test for the proportion of [round/ameboid] vs. [polarized + ramified microglia] between the CTRL or KO group’s untreated control wells and the respective treatment conditions, *p < 0.05, **p < 0.01, ***p < 0.001, and ****p < 0.0001, *n* = 8–11 mice with 952–3,428 cell per group (except: A-804598 in CTRL groups, *n* = 5 mice and 933 cells).

Morphotyping revealed that most microglia were round or ameboid in all conditions and that polarized or ramified phenotypes were rare (at baseline % round/ameboid: CTRL = 78.6%, and KO = 82.6%) (Figures 4A, B). In both groups, stimulation with BzATP and even more stimulation with LPS + BzATP caused a significant increase of round or ameboid microglia and a subsequent decrease of the other two morphotypes. While the increase was similar for the LPS + BzATP condition in both groups, BzATP had a stronger effect in CTRL cells. Both P2X7R antagonists caused a significant reduction of round or ameboid microglia and a gain of polarized and ramified cells in CTRL cultures. Whereas, no relevant changes were found in this respect in the KO groups (Figures 4A, B).

**FIGURE 5:**
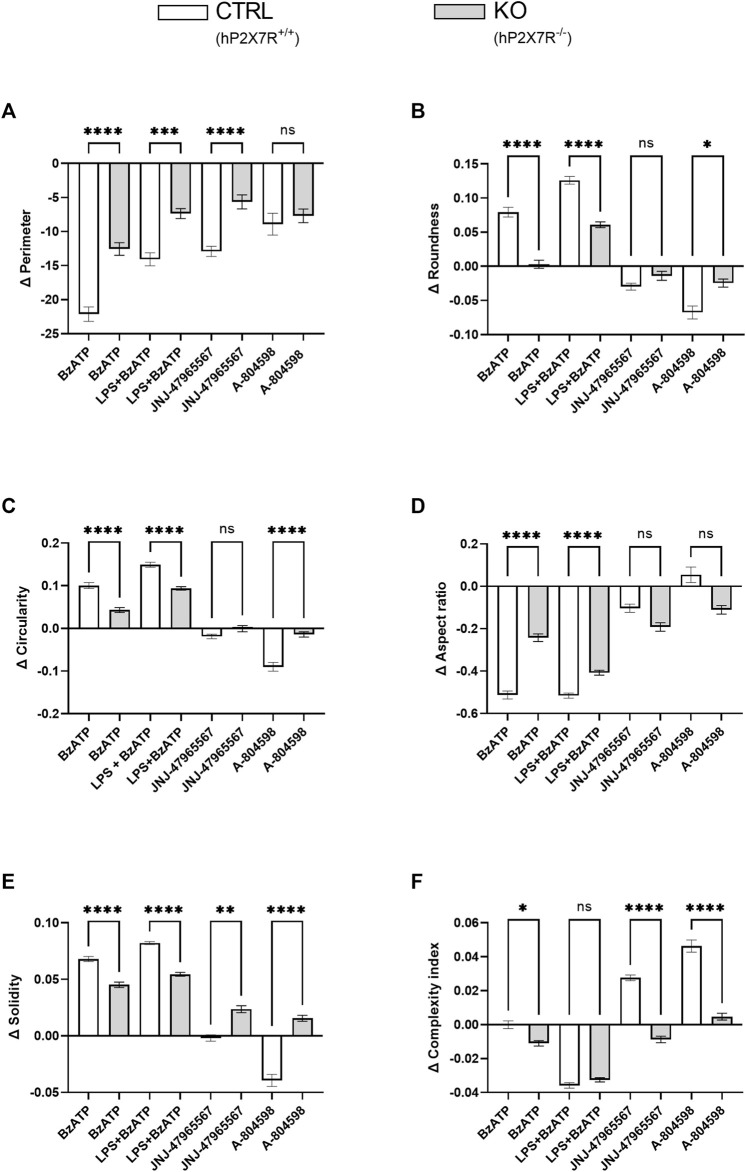
Microglia shape descriptors after hP2X7R-targeting treatments (i.e., BzATP, LPS + BzATP, JNJ-47965567, A-804598). Graphs show between-genotype and treatment comparisons of **(A)** cell perimeter, **(B,C)** roundness and circularity, **(D)** aspect ratio, **(E)** solidity, and **(F)** the complexity index. Shape descriptor values were corrected per group by the respective untreated within-genotype control well means. The hP2X7R-proficient group (CTRL) is displayed in white, the hP2X7R-knockout group (KO) is shown is grey. Data are expressed as mean + S.E.M, KW with Dunn’s test, *p < 0.05, **p < 0.01, ***p < 0.001, and ****p < 0.0001, *n* = 8–11 mice per group (except: A-804598 in CTRL group, *n* = 5 mice). Cells per group (CTRL A-804598): perimeter = 831–2,131 (666); roundness = 815–2039 (645); circularity = 836–2,132 (666); aspect ratio = 785–1997 (616); solidity = 777–2072 (657); complexity = 800–2093 (651).

At baseline, the mean cell perimeter was comparable in CTRL and KO microglia (100 ± 32 µm in CTRLs and 107 ± 37 µm in KOs). Shape descriptor analysis revealed a relative cell perimeter decrease in both genotype groups and in all conditions (Figure 5A). Based on the axiom that microglia activation generally leads to an overall reduction in ramification, this observation is in line with the trend found in the cell area analysis. However, is has to be noted, that the cell area and cell perimeter do not have a simple or linear relation, but very much depend on the experimental condition and the general microenvironment. In agreement with the trend seen in morphotyping, roundness and circularity increase in parallel following BzATP and LPS + BzATP, but decrease after JNJ-47965567 or A-804598 treatment in CTRL cultures. Similar yet less severe dynamics were found in KO cultures (Figures 5B, C). The reverse phenomenon was found with regard to the aspect ratio (Figure 5D). This is not surprising, since roundness and aspect ratio are mathematically related (roundness = 1/aspect ratio). While solidity increased in the CTRL BzATP and LPS + BzATP condition, it remained stable in the JNJ-47965567, and decreased in the A-804598 treated microglia. In KO cultures, on the other hand, the solidity increased in all conditions (Figure 5E). Lastly, the newly devised complexity index was attenuated in response to BzATP and LPS + BzATP in both groups. Surprisingly, the decremental effect of BzATP is decimal in CTRL compared to KO cultures. This difference is driven mainly by the data obtained from a single animal and well, respectively. If removed from the analysis, no difference can be found in the BzATP condition between CTRL and KO cultures. In the P2X7R antagonist treatment, complexity increases in CTRL but not in KO cultures (Figure 5F). In line with the between-genotype findings, within-genotype comparison confirmed hP2X7R-dependent dynamics (Supplementary Figure S4).

### 3.3 Cytokine levels are altered by hP2X7R-tageted stimulation and inhibition

**FIGURE 6:**
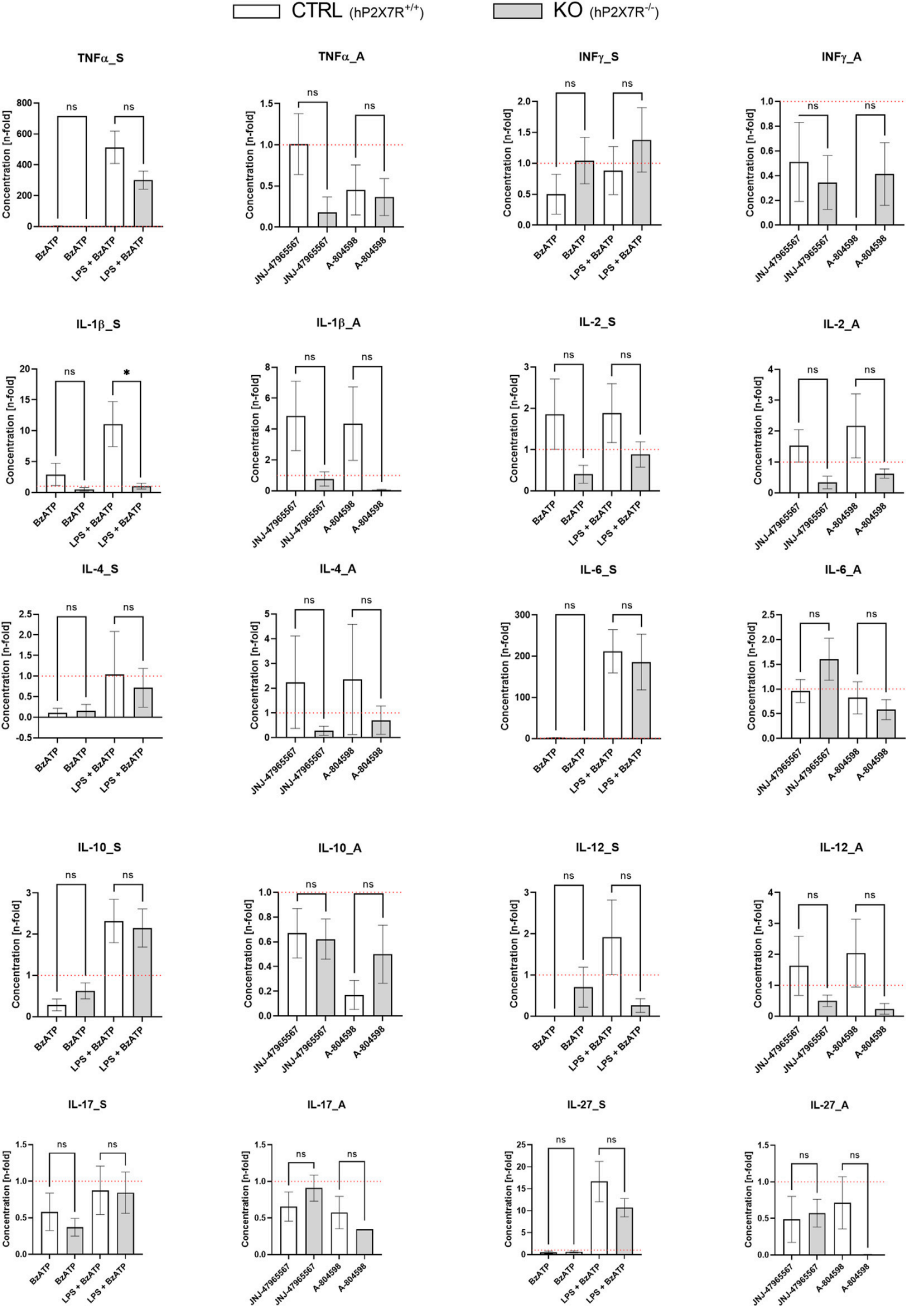
Cytokine secretion alterations following the different hP2X7R-targeting treatments (i.e., BzATP, LPS + BzATP, JNJ-47965567, A-804598). Graphs show the n-fold changes measured in cell culture supernatant of CTRL and KO microglia cultures side by side. For each of the ten cytokines (TNFα, INF-γ, IL-1β, IL-2, IL-4, IL-6, IL-10, IL-12, IL-17/IL-17A, IL-27), a stimulation (_S) and an antagonism (_A) graph is shown. All cytokine levels were first corrected by the untreated within-genotype control well means and n-fold changes were then calculated per group and treatment condition. The hP2X7R-proficient group (CTRL) is displayed in white the hP2X7R-knockout group (KO) is shown is grey. Comparisons were performed between the two genotypes for each of the treatments. Data are expressed as mean + S.E.M, T-test with Welch correction, *p < 0.05, **p < 0.01, ***p < 0.001, and ****p < 0.0001, *n* = 5–7 mice per group.

To study the effects of different immunomodulatory stimuli on microglial hP2X7R-dependant cytokine release, we measured ten cytokines (TNFα, INF-γ, IL-1β, IL-2, IL-4, IL-6, IL-10, IL-12, IL-17/IL-17A, IL-27) in cell culture supernatant using the Luminex^®^ technology (Figure 6). At baseline, cytokine levels between CTRL and KO cultures did not differ significantly. Following stimulation with BzATP, a minor and not statistically significant increase of IL-1β levels was seen in CTRL but not in KO cultures. BzATP also caused a decrease of IL-4 levels (9-fold decrease in CTRL; 6-fold in KO cultures). Changes, however, did not reach statistical significance. After 24 h of priming with 100 ng/mL LPS and 1 h of BzATP, considerable n-fold changes in cytokine levels were detected in both groups for TNFα, IL-6, and IL-27. In this respect, CTRL cultures showed consistently higher levels than KO cultures. The L-1β level increase was significantly different between CTRL and KO cultures (*p* < 0.05). In the P2X7R antagonist conditions, on the other hand, no statistically significant differences were found. Still, a slight trend was observed: in both genotypes treated with either one of the two P2X7R antagonists, the levels of TNFα, INF-γ, and partially IL-6—all highly proinflammatory cytokines–were found to have a mean coefficient of ≤ 1. This equals a drop below within-genotype untreated control well levels, which indicates anti-inflammatory properties of the inhibitors and khP2X7R knockout. Aptly, antagonist application increased IL-4 quantity in CTRL but not KO cultures by 2-fold.

**FIGURE 7:**
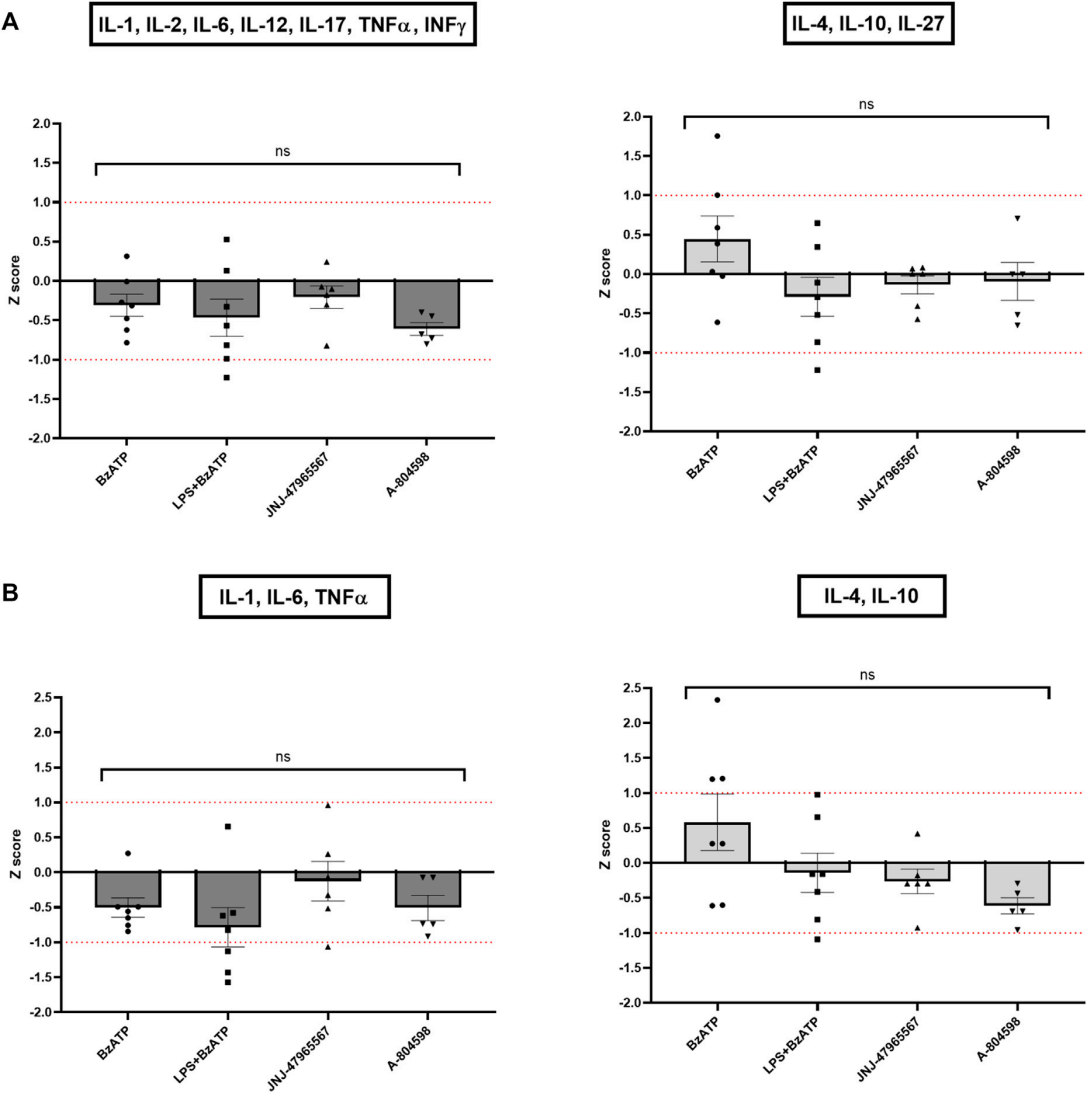
Z scores of pro- and anti-inflammatory cytokine clusters. Pro-inflammatory Z scores are coloured in dark grey, anti-inflammatory ones in light grey. **(A)** Z scores of all pro- or anti-inflammatory cytokines per KO animal and treatment condition in relation to the CTRL sample. **(B)** Z scores of only the core pro- or anti-inflammatory cytokines per KO well and treatment condition in relation to the CTRL sample. Data are expressed as mean + S.E.M, one-way ANOVA with Turkey’s multiple comparisons test, *p < 0.05, **p < 0.01, ***p < 0.001, and ****p < 0.0001, *n* = 5–7 mice per group.

**FIGURE 8:**
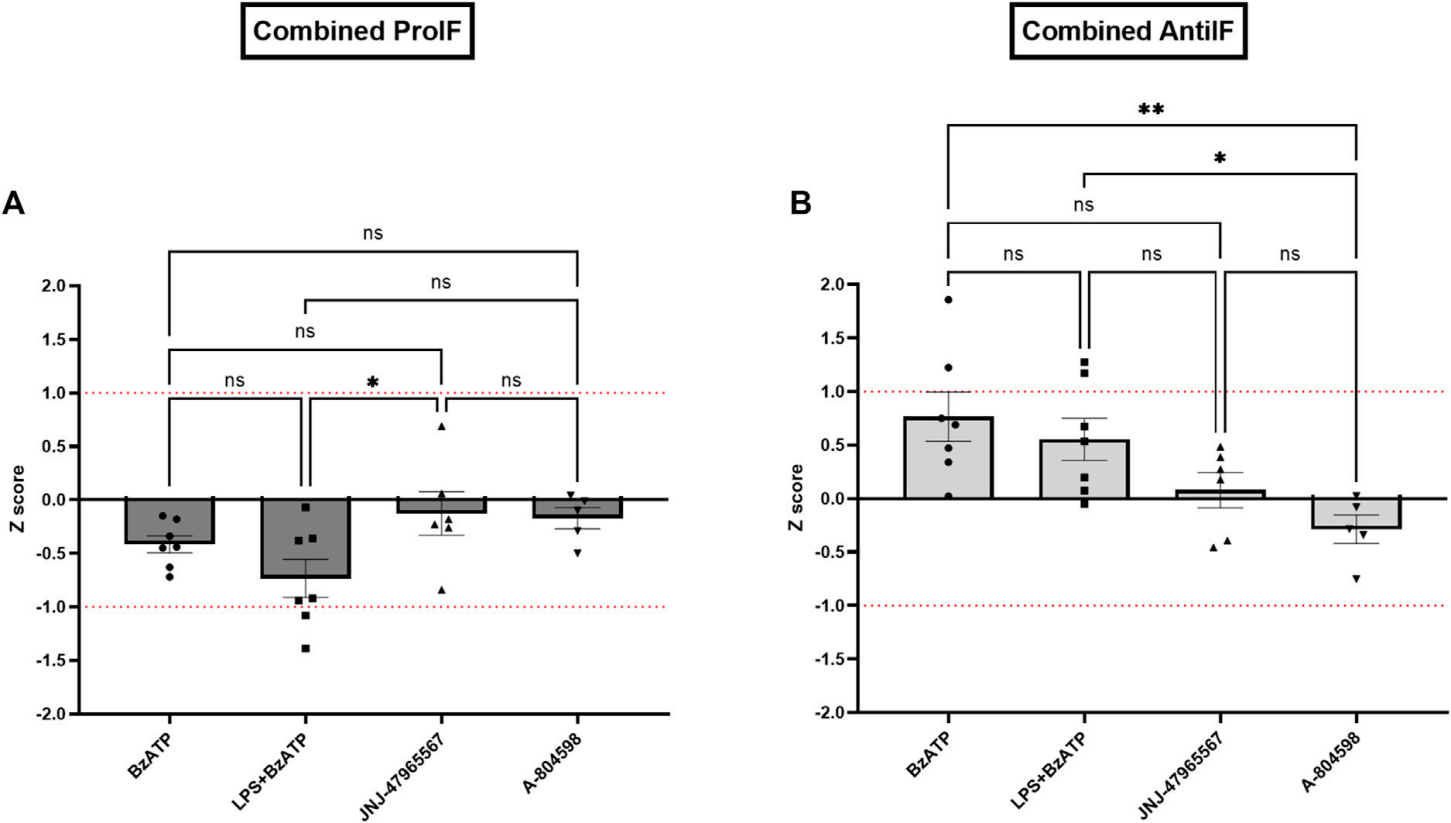
Combined Z scores of **(A)** three core pro-inflammatory cytokines IL-1β, IL-6, and TNFα and microglial roundness (displayed in dark grey) and **(B)** of two core anti-inflammatory cytokines IL-4 and IL-10 and the aspect ratio of microglia per KO animal and treatment condition in relation to the CTRL sample. Data are expressed as mean + S.E.M, one-way ANOVA with Turkey’s multiple comparisons test, *p < 0.05, **p < 0.01, ***p < 0.001, and ****p < 0.0001, *n* = 5–7 mice per group.

In line with the single cytokine results comparing genotypes and treatments, Z score calculations referring to all (Figure 7A) or core (Figure 7B) proinflammatory and anti-inflammatory cytokines confirmed the differences between CTRL and KO cultures: except for the BzATP condition in the anti-inflammatory Z scores, hP2X7R-targeted stimulation led to a weaker increase of pro-inflammatory cytokine cluster in KO compared to CTRL microglia, while hP2X7R-targeting inhibition led to a stronger decrease of the same cytokines in KO compared to CTRL microglia. A corresponding trend was found for the anti-inflammatory Z scores. In the BzATP condition, however, the drop of anti-inflammatory cytokines IL-4, Il-10 ± IL-27 was overall half a standard deviation less in KO compared to CTRL cultures (Figures 7A, B—right). Though not statistically significant, this corroborates the stronger anti-inflammatory mechanisms and pathways in the absence of a functional hP2X7R. The augmented cytokine and shape descriptor Z scores confirmed these observations (Figure 8). For the combined ProIF Z score, a statistically significant difference between KO and CTRLs was found for comparing LPS + BzATP with the JNJ-47965567 condition (*p* < 0.05) (Figure 8A). Similarly, the AntiIF combined Z scores revealed a significant difference for the comparison of the BzATP and LPS + BzATP treatment with the A-804598 condition (*p* < 0.01 and < 0.05, respectively) (Figure 8B). These findings demonstrate the importance of hP2X7R in mediating the effects of both sterile (BzATP) and pathogen plus sterile (LPS + BzATP) stress. The synopsis of the single cytokines and related Z scores with the results of the combined ProIF and AntiIF Z scoring (Figures 6–8) confirms the modulatory role of microglial hP2X7R on a secretory, morphological, and combined level in the context of different immunomodulatory stimuli.

Lastly and on a more general note, measurements revealed low absolute cytokine concentrations in cell culture supernatants with no significant differences between CTRL and KO cultures at baseline (Supplementary Figure S5).

## 4 Discussion

To further characterize the role of microglial hP2X7R signaling in mediating the cellular and humoral response following stimulation or selective P2X7R antagonism, we have used fluorescence-based single cell morphotyping as well as cytokine measurements in primary murine microglia cultures. The novelty of our approach lies in the use of a genetically engineered mouse model possessing a humanized P2X7R together with detailed morphological analyses and measurements of so far unchartered cytokines. This combinatory method allowed us to generate meaningful and new insight into hP2X7R properties and the consequences of its absence in different treatment conditions, which is vital for further *in vivo* and clinical studies. We were able to demonstrate that hP2X7R influences microglia morphology as well as cell count, area, perimeter, roundness, circularity, aspect ratio, solidity, and complexity following stimulation as well as selective antagonism. Cytokine quantification confirmed the effects of hP2X7R, primarily following stimulation *via* LPS and/or BzATP, in the form of TNFα, IL-1β, IL-6, and IL-27 level increase and IL-4 level decrease. To the best of our knowledge, this is the first detailed *in vitro* characterization of both morphological and cytokine changes in hP2X7R-expressing microglia after selective immune stimulation or inhibition.

In contrast to the *in vivo* situation (Kozlowski and Weimer, 2012; Leyh et al., 2021), we found microglial cell area to be increased in both genotypes. In addition, KO microglia were about 25% larger than WT cells at baseline. The mean microglial cell perimeter was, however, relatively low compared to the *in vivo* situation. Our findings are, though somewhat different from the *in vivo* condition, in line with previous publications evaluating microglial morphology in primary murine cell cultures (Caldeira et al., 2014). The observed combination of an increased cell area and a small perimeter together with the high proportion of round or ameboid microglia at baseline indicate that our *in vitro* microglia are somewhat activated compared to resting *in vivo* cells. Whether the observed morphological observation is exclusively indicative of a rather proinflammatory M1 polarization remains unclear. It has been shown that, compared to naive cells, a higher cell area is associated with a more M2-like phenotype, while a smaller cell area is commonly found in M1 polarized macrophages (Rostam et al., 2017). Whether this observation also transfers to murine microglia, especially in the *in vitro* situation, is not known. The classical ameboid morphology of microglia is however, at least *in vivo*, associated with an increased phagocytotic capacity, proinflammatory cytokine release, and therefore M1 polarization (Tam and Ma, 2014). Aptly, immunohistochemistry-based machine learning approaches have shown that ameboid microglia (i.e., activated morphotype) are associated with a small cell area and perimeter as well as an increased solidity in the murine cortex and hippocampus (Leyh et al., 2021). Since the employed procedure of microglia isolation from neonatal mice as well as the *in vitro* culturing is unphysiological and thus a strong contextual stimulus, observing morphologically activated microglia (i.e., round or ameboid) is not surprising and must be considered an inherent methodological drawback of primary cell cultures, especially in serum-containing growth medium (Montilla et al., 2020). In line with this argument and similar to our findings, previous studies that have made use of serum-containing medium have shown that microglia lose the majority of their ramifications *in vitro* (Caldeira et al., 2014; He et al., 2017; He et al., 2021; Hu et al., 2022). This phenomenon of “deramification” can be partially prevented with a serum-free growth medium, which contains TGF-β2, cholesterol, and macrophage colony-stimulating factor (TIC factors) to sustain microglia survival (Bohlen et al., 2017; Montilla et al., 2020). Some scholar have even argued that TIC medium is closer to the *in vivo* and thus physiological condition (i.e., the cerebrospinal fluid), since it contains only low protein and bioactive factor levels (Montilla et al., 2020). In opposition to this argument we reason, that both the serum-containing as well as TIC medium have inherent advantages and drawbacks. For instance, while serum-containing growth medium sustains and simultaneously activates cultured microglia, the serum-free TIC medium of Bohlen et al. (2017) artificially antagonizes the effects of the isolation procedure and culturing condition by forcing cells back into a more ramified and less activated state using TGF-β2. This TIC environment might bias experiments on inflammatory properties of microglia. Both methods are thus, by definition, non-physiological conditions and therefore skew observations in one or the other way (Montilla et al., 2020). However, if factoring in the methodological drawbacks into the experimental framework and data interpretation, both approaches can yield meaningful results. Since our primary study objective was to characterize the inflammatory effects of microglial hP2X7R under different conditions *in vitro*, an anti-inflammatory environment in the form of the TIC growth medium was deemed unsuitable. This decision was further corroborated by findings from *in vitro* experiments with serum-containing medium, which have shown that method-related inflammation drops significantly after 10 days *in vitro* (Caldeira et al., 2014).

As to be expected from prior studies, our morphotyping and shape descriptor analysis revealed that most microglia were rather large and round with little to no ramifications in both genotypes and all conditions. Irrespective of this clearly shifted morphological baseline, we were able to identify a distinct and, compared to CTRL cells, hP2X7R-potentiated increase in morphological and secretory activation towards a more M1 polarized phenotype (i.e., increase in round or ameboid cells, decrease in area and perimeter, increase in roundness/circularity and a decrease in aspect ratio, increase in solidity, reduction of complexity, increase of CD86 intensity in the LPS + BzATP condition, inflammatory cytokine release including IL-1β). This agrees with findings from earlier studies, which have found IL-1 β expression to be associated with a proinflammatory morphotype including a decrease in cell area and perimeter as well as an increase in circularity (Fernández-Arjona et al., 2019). Our findings further agree with the triangle TNFα, IL-1β, and IL-6, which is the typical secretory cluster seen in M1 polarization (Orihuela et al., 2016). This becomes even more evident when considering the cytokine dynamics beyond TNFα, IL-1β, and IL-6, namely, the decrease of the anti-inflammatory IL-4 and IL-10 following BzATP in both genotype groups as well as the increase following antagonist application in CTRL cells. IL-4 has a strong anti-inflammatory effect on microglia, triggers M2 polarization, and promotes neuronal stem cell survival and proliferation (Zuiderwijk-Sick et al., 2021; Jiang et al., 2022). This combination of P2X7R amplified humoral inflammation and morphological activation and, vice versa, P2X7R inhibition induced anti-inflammatory cytokine secretion and a trend towards a resting morphotype emphasizes the role of hP2X7R in inflammation regulation. This is highly important, since P2X7R has been hypothesized to mediate psychosocial stress induced neuroinflammation and ultimately depression and depressive-like behaviours, respectively (von Mücke-Heim and Deussing, 2022; von Muecke-Heim et al., 2021). Accordingly, clinical trials have demonstrated the effect of gain-of-function single nucleotide polymorphisms (SNPs) on depression (Andrejew et al., 2020; Ribeiro et al., 2019a; Urbina-Treviño et al., 2022; von Mücke-Heim and Deussing, 2022), which fits the hypothesis of an increased immune activation along with defective inflammation resolution mechanisms in depression (Debnath et al., 2021). Our cytokine findings are furthermore in line with the well-known connection between toll-like receptor mediated LPS effects in pro-IL-1β production and P2X7R induced caspase-1 activation in amplifying mature IL-1β release (Ferrari et al., 2006; Mingam et al., 2008). Also, the shift towards ameboid morphology and elevated cytokines TNFα, IL-1β, IL-6 were found in related studies using LPS + BzATP (de Torre-Minguela et al., 2016; He et al., 2021). These findings are also conceptually in agreement with studies using BzATP and LPS as well as P2X7R antagonists *in vivo* and *in vitro* (Andrejew et al., 2020; Ribeiro et al., 2019a; Ribeiro et al., 2019b; von Muecke-Heim et al., 2021). Accordingly, and beyond these well-known proinflammatory effects, we were able to identify an increase of complex phenotypes (i.e., polarized and ramified microglia, reduction in roundness and circularity and solidity, increased complexity) as well as a tendential decrease in inflammatory cytokines after JNJ-4796556 and A-804598 application. Cluster findings in the form of cytokine and combined cytokine-morphology Z score analyses confirmed the findings from morphotyping, shape descriptor extraction, and single cytokine comparison. With regard to the observed microglia area and perimeter dynamics in CTRL microglia, we argue that stimulation caused the cells to be activated and to polarize towards a small and ameboid morphotype, while P2X7R antagonism resulted in a similar yet less pronounced trend without the increase of round or ameboid cells. Based on the evidence presented above and considering the shifted baseline and morphotyping results, respectively, the cell area and perimeter decrease in stimulation conditions likely equals a trend towards M1-like activation, while the P2X7R antagonism resembles a trend towards polarized or rod-like cells and thus more M2-like microglia. This aligns with findings from prior studies, which have shown that M2 microglia exhibit an elongated morphology (i.e., rod-like/polarized morphology) and even express typical M2 surface markers accompanied by a reduced secretion of inflammatory cytokines when forced in a polarized shape (McWhorter et al., 2013). And even though the concept of M1 and M2 polarization has been criticized for being a too simplistic approach towards microglia phenotypes (Ransohoff, 2016), it provides a useful framework of activation with two maxima. The latter is a helpful tool to dissect inflammation in an experimental setting (Orihuela et al., 2016; Jurga et al., 2020). Keeping this and all the observed morphological and secretory phenomena in mind, our data demonstrate the effect of hP2X7R on microglia on the continuum between ramified or resting, rod-like or polarized, and round or ameboid microglia (Tam and Ma, 2014; Jurga et al., 2020). It has, however, also been demonstrated that P2X7R is part of inflammation resolution *via* pathways other than NLRP3 and NFκB signaling in resting and M2 macrophages (de Torre-Minguela et al., 2016). This highlights the complexity of interpreting P2X7R-related dynamics in cell culture as well as in specific diseases and inflammatory stages (von Mücke-Heim and Deussing, 2022), which also relates to our findings. However, though we consider our findings overall conclusive, especially taking into account the detailed morphological analyses, our cytokine data have limitations with regards to the sample size and the multiplex analyte kit used for cytokine measurements. Therefore, we would suggest future studies to increase the sample size and to employ high-sensitivity ELISA-based assays for quantification of cytokines combined with intracellular pathway analyses (e.g., NFκB or PPAR δ) to best determine the role of hP2X7R in M1 and M2 polarization (Wang et al., 2014). Though it is established that antigen-presenting cells including microglia secret IL-27 (Kawanokuchi et al., 2013), this is, to the best of our knowledge, the first report of hP2X7R changed IL-27 secretion in the context of microglia and hP2X7R. Though IL-27 was initially classed as proinflammatory by promoting TNFα release and its relation with IL-6 signaling (Yoshida and Hunter, 2015), novel evidence has highlighted its function as a negative regulator of Th1, Th2, Th9, and Th17 function (Kawanokuchi et al., 2013; Yoshida and Hunter, 2015). Novel results even indicate a role of IL-27 in increasing T_reg_ function (Yoshida and Hunter, 2015; Le et al., 2020), which suggests a role of IL-27 in inflammation resolution (Yoshida and Hunter, 2015; Lalive et al., 2017). This function of IL-27 is highly interesting in the context of the emerging evidence on immunometabolic subtypes and related phenomena in depression (Milaneschi et al., 2020; von Mücke-Heim and Deussing, 2022). Several studies have demonstrated altered T cell function in depression, particularly an increase in Th17 and a decrease in T_reg_ cells (Miller, 2010; Suzuki et al., 2017; Ellul et al., 2018; Moschny et al., 2020). Surprisingly, IL-27 has not been extensively investigated in depression and does therefore not surface in large metanalyses on cytokine alterations in depression (Osimo et al., 2020). Ultimately, our findings underline the complex functions of microglial hP2X7R in inflammation downstream of sterile and combined immune stimuli and single out the potential connection between microglial hP2X7R and IL-27. The relevance of our findings remains to be determined. Thus, further trials are needed to investigate this potential role of P2X7R in altering microglia-derived IL-27 secretion patterns. The latter might be particularly relevant in the context of inflammation resolution of mood disorders like depression *via* treatments like electroconvulsive therapy, which is known to a cause short-term spike yet long-term attenuation of systemic and CNS inflammation (van Buel et al., 2015a; van Buel et al., 2015b; van Buel et al., 2017).

Taken together, we have demonstrated that microglial hP2X7R causes and potentiates morphological and secretory changes downstream of targeted immune stimulation as well as inhibition. In the future, additional *in vitro* and *in vivo* studies are necessary to further clarify the contribution of P2X7R-related purinergic signaling to mood disorder genesis and therapy. These studies should focus on immune changes downstream of differential gene-environment interactions (i.e., hP2X7R expression and function x psychosocial and/or pathogen-derived stressors), since the corresponding patterns, which are currently largely unchartered, likely contribute to state- and subtype-specific mood disorder immune pathology.

## Data Availability

The original contributions presented in the study are included in the article/Supplementary Material, further inquiries can be directed to the corresponding author.
