# Supplementary material for: The human P2X7 receptor alters microglial morphology and cytokine secretion following immunomodulation
Source: Front Pharmacol. 2023 Apr 10;14:1148190. doi: 10.3389/fphar.2023.1148190 (PMC10123291; doi:10.3389/fphar.2023.1148190)
Supplement: Supplementary file 1 [file DataSheet1.pdf]

Supplementary Material

The human P2X7 receptor alters microglial morphology and cytokine secretion following immunomodulation

Iven-Alex von Mücke-Heim, Jana Martin, Manfred Uhr, Clemens Ries, Jan M. Deussing

* **Correspondence:** Jan M. Deussing: deussing@psych.mpg.de

Supplementary Figures

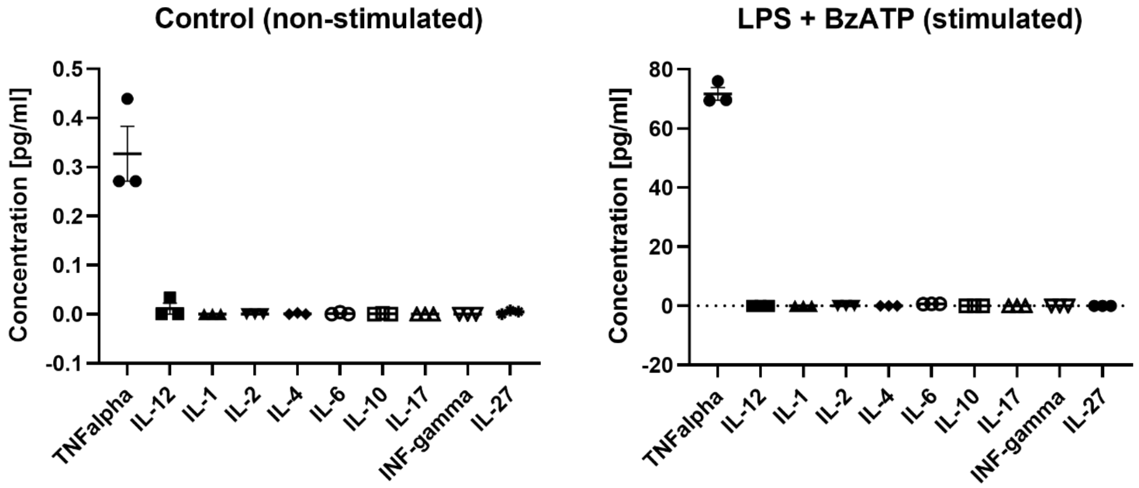

Supplementary Figure 1

Validation of the Luminex® Discovery Assay's reliability. Triplicate measurements of unstimulated KO and stimulated CTRL microglia (one well each) confirmed assay reliability and validity in different microenvironments.

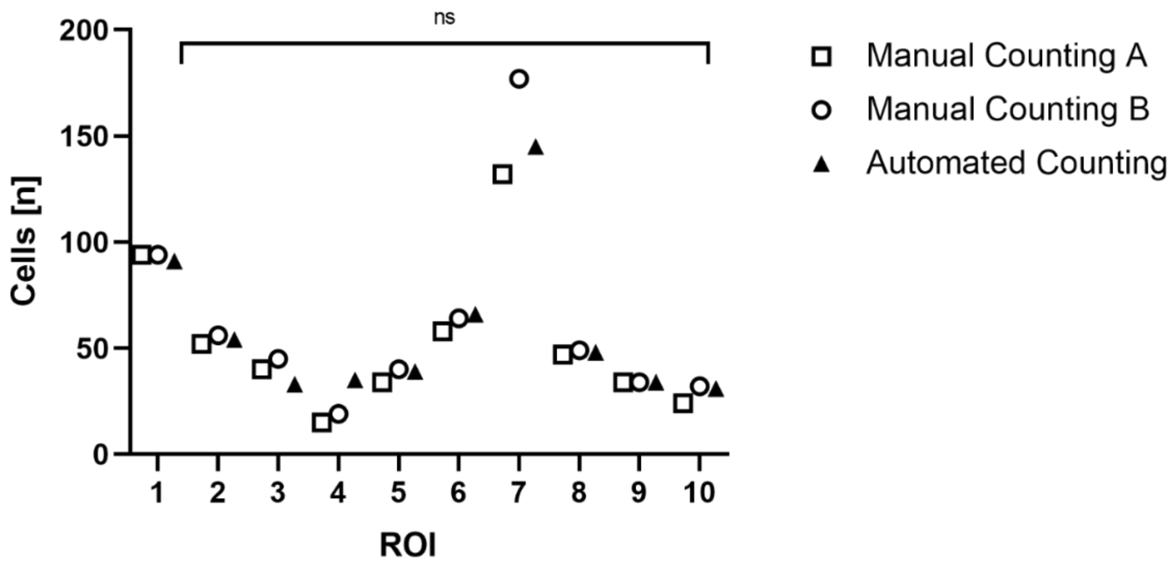

Supplementary Figure 2

Validation of the custom-designed Fiji cell counting macroinstruction. For the validation, n = 10 ROIs were selected at random to avoid bias, especially selection and confirmation bias. Manual quantification was performed by two independent experimenters, which were also blinded for the automated results. The experimenters counted double positive cells (Iba1/GFP and DAPI/red), while the Fiji macro registered only DAPI/red nuclei. Results of cells per ROI were highly similar between the manual and automated results. Summary statistics revealed no significant difference between the groups (T test for A vs. B, A vs. automated counting, and B vs. automated counting), *p < 0.05, **p < 0.01, ***p < 0.001, and **** p < 0,0001.

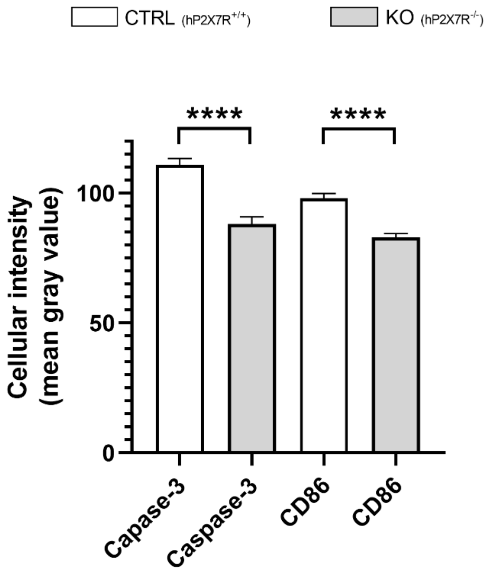

Supplemenatry Figure 3.

Intensity differences in LPS+BzATP treated microglia. In LPS+BzATP stimulated wells, cellular intensities were significantly higher in CTRL than in KO microglia for both cleaved caspase-3 and CD86. Data are expressed as mean ± S.E.M, T test with Welch correction, *p < 0.05, **p < 0.01, ***p < 0.001, and **** p < 0,0001, n = 2 mice.

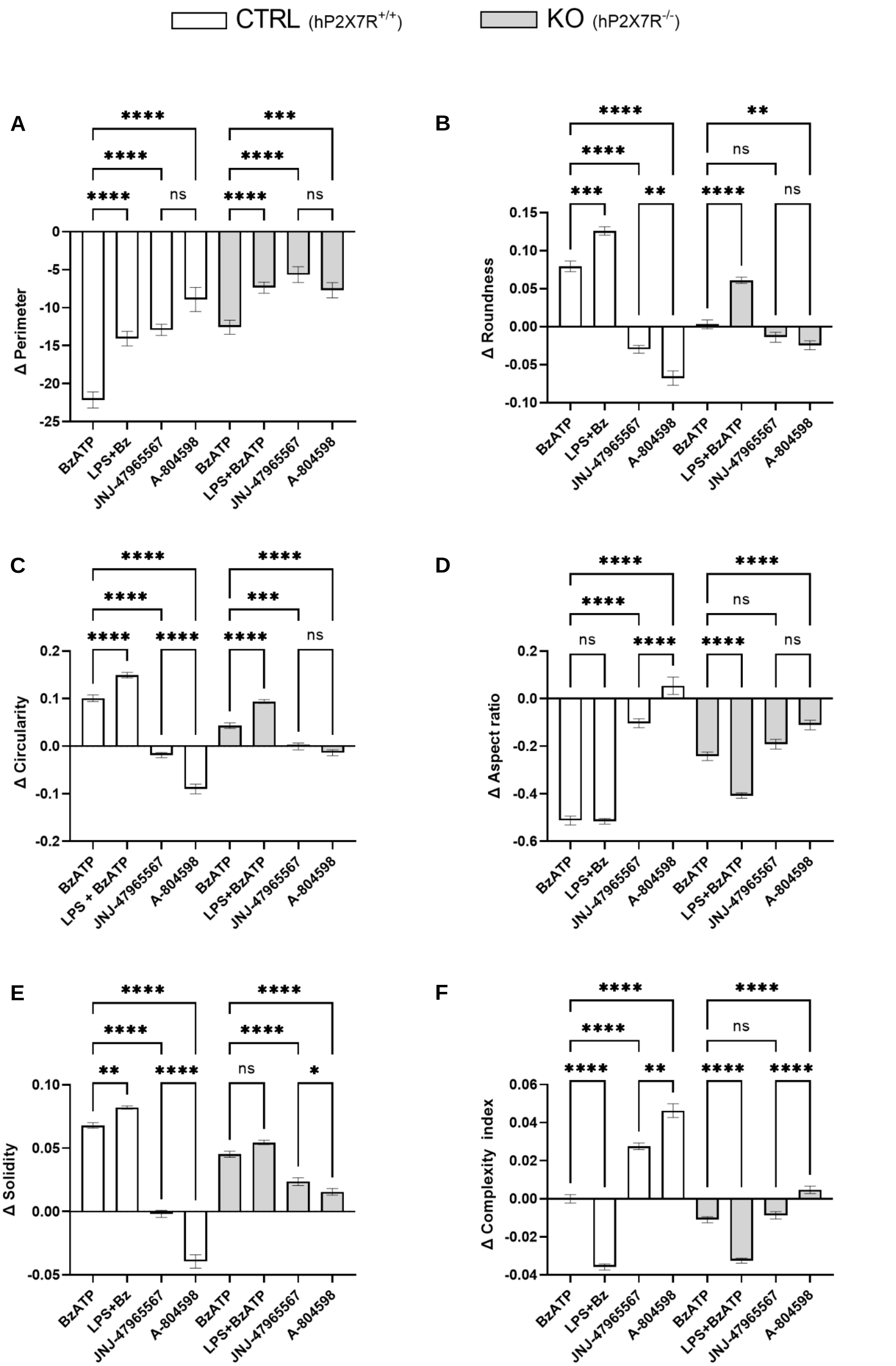
CTRL (hP2X7R+/+)
KO (hP2X7R-/-)
A
Δ Perimeter
B
Δ Roundness
C
Δ Circularity
D
Δ Aspect ratio
E
Δ Solidity
F
Δ Complexity index
BzATP
LPS+Bz
LPS + BzATP
LPS+BzATP
JNJ-47965567
A-804598
ns

Supplementary Figure 4

Within-genotype comparison of shape descriptors confirms the effects of P2X7R-targeted stimulation or inhibition. (**A-F**) In both CTRL and KO microglia, comparison of the BzATP condition with LPS+BzATP and the two selective P2X7R antagonists confirmed the results from between genotype-comparison. Interestingly, JNJ-47985567 and A-804598 application had a varying effect on microglial shape descriptors within the CTRL an KO group. This suggests either relevant pharmacodynamic differences between the two or pleiotropic off-target effects, or both. Data are expressed as mean ± S.E.M., KW with Dunn's test, *$p < 0.05$, **$p < 0.01$, ***$p < 0.001$, and **** $p < 0,0001$, n = 8-11 mice per group (A-804598 in CTRL group, n=5).

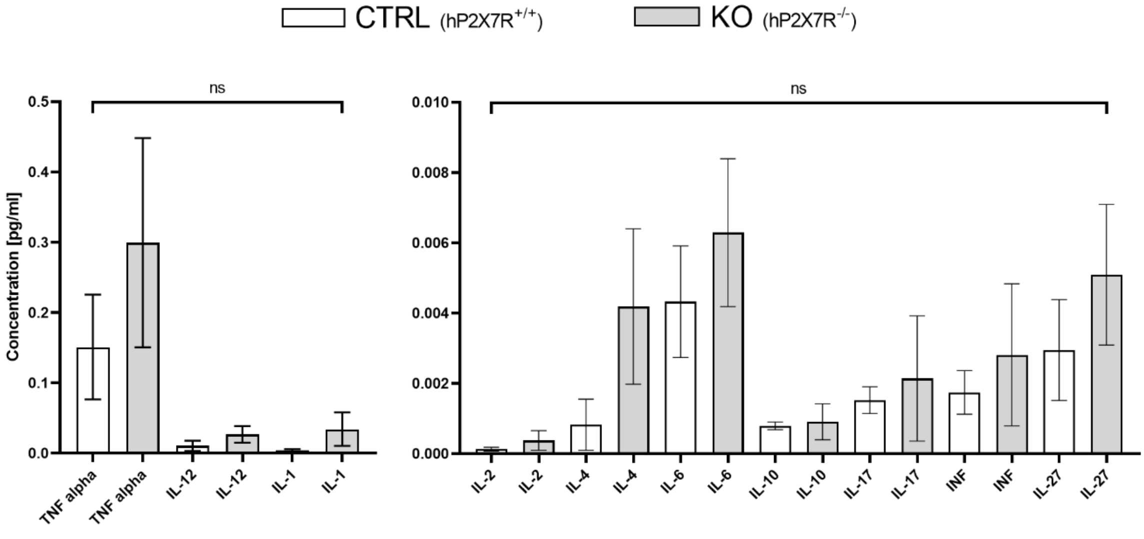

Supplementary Figure 5

Absolute cytokine levels at baseline. In both CTRL and CO microglia, cytokine levels were low. Comparison between the genotypes revealed no difference between pooled, untreated control wells (baseline). Data are expressed as mean ± S.E.M, KW with Dunn's test, *p < 0.05, **p < 0.01, ***p < 0.001, and **** p < 0,0001, n = 5-7 mice per group.
